# Initial experience with novel CGRP-receptor inhibitor therapy in Migraine in the United Arab Emirates: a retrospective observational study

**DOI:** 10.1186/s12883-021-02507-y

**Published:** 2021-12-14

**Authors:** Bui Bao Khanh Dinh, Waseem Hamed Aziz, Alessandro Terruzzi, Derk Wolfgang Krieger

**Affiliations:** 1 Mediclinic Dubai, Institute for Neuroscience, Dubai, United Arab Emirates; 2 Martin-Luther University Halle-Wittenberg, Universitätsplatz 10, 06108 Halle (Saale), Germany; 3 Mohammed Bin Rashid University of Medicine and Health Sciences, Dubai, United Arab Emirates

**Keywords:** Migraine headache, CGRP inhibitor, Erenumab, Retrospective study

## Abstract

**Background:**

Erenumab is a calcitonin gene-related peptide (CGRP)-receptor antibody inhibiting CGRP function. CGRP is prominently involved in the pathophysiology of migraine through nociceptive modulation in the trigeminovascular system. This study aims to explore the treatment effect of erenumab in a real-life setting.

**Methods:**

In this retrospective observational study, we analyzed the data of 91 patients with migraine receiving at least three consecutive monthly injections of erenumab and followed up for 3–12 months. The primary objective was to describe the reduction in monthly migraine days throughout the follow-up period. To identify patients who responded to treatment, we analyzed the association between different patient characteristics and their treatment outcomes.

**Results:**

Seventy-three patients (80.2%) responded to erenumab treatment, defined as ≥50% reduction of migraine days per month, across all migraine types. It was noted that ethnicity (*p*-value = 0.015) and older age (p-value = 0.035) were associated with clinically relevant improvement of symptoms. Middle Eastern ethnicity was related to less improvement of symptoms while Europeans were more likely to benefit from erenumab therapy (odds ratio: 12.788, *p* = 0.037). Patients aged from 31 to 40 and 41–65 years benefited most from erenumab treatment with a response rate of 77.8 and 89.9%, respectively, also confirmed by logistic regression (*p* = 0.047). Neither gender nor dose increase of erenumab showed association with the reported clinically relevant improvement of the symptoms. An association between clinically relevant improvement of headaches and the type of migraine was also noted. Around 87.9% of patients with episodic migraine responded to treatment, followed by 84.1% of chronic migraine patients and 50% of medication overuse headache patients. Medication overuse headache showed a lower probability of therapy success with erenumab (odds ratio: 0.126, *p* = 0.039). An improvement of headaches was eminent in patients who received 140 mg erenumab monthly (2 × 70 mg injections) and patients who had one injection every two weeks.

**Conclusions:**

Erenumab is a novel preventive treatment for all migraine types. Clinically relevant improvement of headaches and reduction of monthly migraine days were demonstrated in patients that continued the treatment course. In real-life, a substantial number of patients suspended therapy early, reasons for which need further investigation.

## Introduction

Migraine is a common neurologic condition with important consequences in terms of loss of productivity and quality of life [1]. Migraine represents the second leading cause of disability worldwide and the first cause in young women when measured by disability-adjusted life years (DALYs) [2]. As biomarkers are lacking, the diagnosis of migraine is established by clinical investigation and careful history (ICHD-3 classification) [3]. Migraine can be traced back to childhood; however, it is most commonly diagnosed in adolescents and young adults. Women are 3 times more often affected by migraines than men. The high prevalence of migraine among women is between 20 and 30%. The intensity and duration of symptoms may vary individually but usually follows a stereotype course [3].

Migraine is defined by episodes of head pain that are often described as throbbing and almost exclusively unilateral. Typically, headaches are accompanied by photophobia, phonophobia, neck pain, and fatigue [3]. Migraine with aura makes up to one third of all cases. Chronic migraine is defined by ≥15 headache days per month and has a prevalence of 1–2% in the general population. Episodic migraine are characterized by less than 15 headache days per month [4]. Medication-overuse headache is present in almost 1% of the general population and is defined by ≥15 headache days per month for more than 3 months associated with the overuse of painkillers [5].

Treatment of migraine requires a multi-disciplinary approach to mitigate triggers through lifestyle changes using medications as well as alternative therapies including acupuncture, counseling, exercising, mindfulness, and meditation [3]. To obtain satisfactory results, patients need careful neurologic assessment, education, and a clear treatment plan that empowers the patient and ensures adherence to treatment. In migraine with infrequent attacks; paracetamol, non-steroidal anti-inflammatory drugs (NSAIDs), triptans, and antiemetics are recommended medical therapies. In episodic and chronic migraines with at least 2 attacks per month, preventive medication is recommended. Many repurposed medications, such as beta-blockers, are prescribed off-label for the prevention of migraine. However, various side effects may reduce the adherence to these medications. Therefore, the development of drugs with specific targets in the pathophysiology of migraine and accepted safety profiles has been crucial [3, 6]. In 1990, Goadsby et al., observed a potential association between migraine attacks and calcitonin gene-related peptide (CGRP) release [7]. CGRP is a peptide neurotransmitter that has several impacts on the central nervous system. CGRP was described as a crucial mediator in the process of a migraine attack with potential therapeutic implications. CGRP induces a trigemino-neurovascular system trigger which subsequently produces a migraine phenotype. It was also revealed that CGRP is released during acute migraine attacks [8]. An early CGRP inhibitor prototype was abandoned due to its toxic profile [9]. As the trigeminal nerve ganglion and adjacent dura are not isolated by the blood-brain-barrier, they can be targeted by peripherally acting antibodies. Anti-CGRP and anti-CGRP receptor antibodies showed significant effects on the course of migraine [8]. Since then, monoclonal antibodies were engineered to either directly bind to CGRP or block their receptors [8].

Following pivotal randomized trials, CGRP inhibitors were approved for patients with all types of migraine and medication overuse headache [10]. Four monoclonal antibodies are currently approved for episodic and chronic migraine [6]. Besides erenumab, the first drug approved, other anti-CGRP monoclonal antibodies, such as galcanezumab, eptinezumab, and fremanezumab have been studied [11, 12].

This study aims to investigate the use of CGRP receptor monoclonal antibody erenumab. In a period of 6 months after erenumab’s approval in the United Arab Emirates (UAE), we selected patients who were initiated on erenumab and were previously naïve to other anti-CGRP ligand or receptor treatments. Patients with all migraine types were included in this study. All enrolled patients had subjectively severe or frequent migraine headaches despite previously on prevention treatment with repurposed medications.

## Methods

### Study design

This retrospective observational study was conducted at the Headache Clinic of Mediclinic City Hospital, which serves as a tertiary hospital in Dubai. The study’s protocol was reviewed and approved by the Dubai Healthcare City Authority – Regulatory Review Ethics Committee (DHCR Rec 2021 10 MCH Migraine). Informed consent was waived by the Dubai Healthcare City Authority – Regulatory Review Ethics Committee (DHCR Rec 2021 10 MCH Migraine) as it was not required due to the retrospective nature of the study and considering that there was no associated risk.

**Fig. 1:**
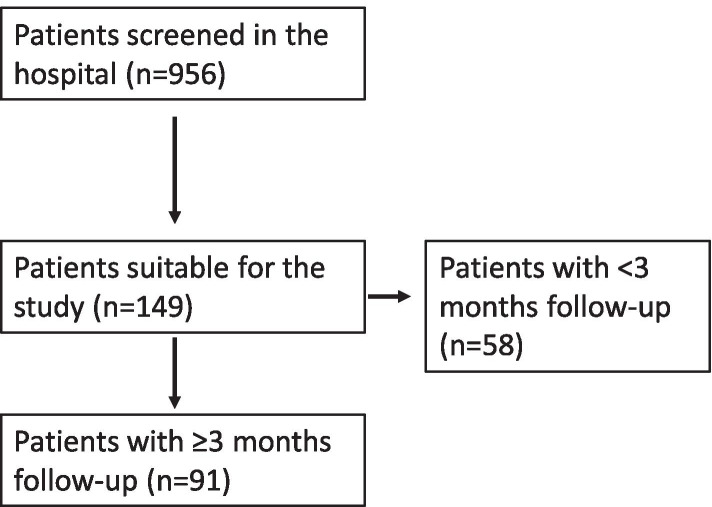
Flow chart patient recruitment. In this study 956 patients were observed. Patients who meet the criteria of the ICHD-3 classification for migraine were included (*n* = 149). For further analysis, we excluded the patients who did not meet the study requirements of at least 3 consecutive monthly injections of erenumab over ≥3 months of follow-up (*n* = 58). The final cohort included 91 patients

In this study, we extracted the data from the patient files of 956 subjects with migraine, of whom 149 were treated with erenumab (Novartis International AG, Basel, Switzerland) for the first time. Patients who met the criteria of the ICHD-3 classification for migraine were included. A recruitment period was set to review the performance of patients who were treatment-naïve for erenumab but received other preventive therapies before. Initially, 149 patients were identified, included and their data was analyzed. However, 58 patients were excluded from analysis as they did not meet the study requirements of at least 3 consecutive monthly injections of erenumab. Ninety-one patients were included in the final analysis, as seen in fig [1].. Patients were recruited, followed, and presented in this study for a period of 6 months after the approval of erenumab in the UAE. Erenumab dose was increased to 140 mg in a subset of patients as per the decision of the treating physician. We analyzed the association between different patient characteristics and their treatment outcomes to predict patients’ response to treatment. Response rate was defined as the reduction of monthly migraine days by at least 50% in all migraine types. We also reported the number of patients discontinuing therapy and the reasons for discontinuation.

### Data collection

Patients were seen and followed up by consultant neurologists at Mediclinic City Hospital Dubai. Collected patient data included gender, age, ethnicity, type of migraine, previous years of migraine, monthly migraine days in the 2 months prior to erenumab initiation and in the 3 months after initiation, a dose increase of erenumab (70 to 140 mg during the treatment), and the duration of follow-up. Migraine subtypes were classified according to ICHD-3 classification into episodic, chronic, and medication overuse headache migraines. Only patients with ≥3 months of follow-up were included in this analysis. The severity of symptoms was collected 2 months before baseline and for at least 3 months after erenumab initiation. All symptoms were extracted from electronic medical records.

### Data analysis

Data for gender, age, ethnicity, and type of migraine were analyzed for basic descriptive statistics, frequency of events, mean and standard deviation. The Chi-squared test was used to assess the treatment effects over groups. Significant results were further analyzed and confirmed by Logistic Regression in SPSS. The paired student’s t-test was used to determine differences in sequential follow-up of migraine days per month. The alpha significance level was set at 5%. Since primary endpoints are descriptive in nature, sample size calculations were not performed, and all summary metrics are reported based on the available analyzable patient data based on our study requirements. All analysis of data was performed with SPSS version 26.0.0.0 (IBM Corp., Chicago, IL) and Prism (GraphPad Software Inc., San Diego, CA). Boxplots were used to illustrate the effect of erenumab treatment on migraine days.

## Results

In this study, we analyzed 91 patients who were treated with various preventive therapies before initiation of monthly erenumab injection (Fig. 1).

An overview of the patient characteristics is shown in table [1]. The majority of patients were females (85.7%) and between 30 and 65 years of age (82.4%). The mean age was 38.7 ± 9.5 years and 85.7% were females. All patients either did not respond or could not adhere to their previous prevention regimens. Among the 91 patients with migraine, 33 were previously treated with antidepressants, 28 with anticonvulsants, 16 with botulinum toxin, and 8 with beta-blocker. The majority of patients were originally of Middle Eastern descent (49.4%), followed by Asian, European, and other ethnicities. Chronic migraine was the most commonly presented type of migraine in this study (48.3%). Around 65.9% of the patients reported a migraine history of more than 20 years (Table 1).

**Table 1:** Baseline characteristics of the patients

Characteristic	Number of patients (%) *n* = 91
Gender	
Female	78 (85.7%)
Male	13 (14.3%)
Range of age	
0–20	2 (2.2%)
21–30	14 (15.4%)
31–40	27 (29.7%)
41–65	48 (52.7%)
Ethnicity	
Middle East	45 (49.4%)
Asian	17 (18.7%)
Europe	20 (22.0%)
Rest of the world	9 (9.9%)
Previous years of migraine	
0–19 years	31 (34.1%)
≥20 years	60 (65.9%)
Type of migraine	
Medication overuse headache	14 (15.4%)
Episodic migraine	33 (36.3%)
Chronic migraine	44 (48.3%)
Reduction of migraine days in all migraine types	
0–24%	13 (14.3%)
25–49%	5 (5.5%)
50–74%	10 (11.0%)
75–100%	63 (69.2%)
Dose increase of erenumab to 140 mg	
Yes	14 (15.4%)
No	77 (84.6%)

Analysis of the individual reduction of migraine days is depicted in table [1]. Thirteen patients (14.3%) had a 0–24% reduction of monthly migraine days. Five patients (5.5%) had a 25–49% reduction, 10 patients (11.0%) had a 50–74% reduction, and 63 patients (69.2%) had a 75–100% reduction of migraine days per month.

**Fig. 2:**
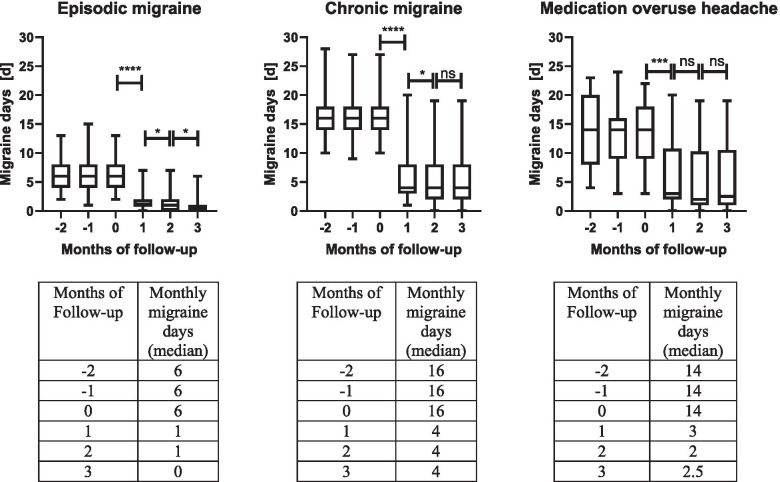
Median monthly migraine days during follow-up. The migraine days were documented 2 months before the initial erenumab treatment and 3 months after. The patients showed in all migraine types after first injection a significant reduction of the monthly migraine days (* *p* < 0.05, ** *p* > 0.01, *** *p* < 0.001, **** *p* < 0.0001)

For the episodic migraine patients, the mean of monthly migraine days before treatment was 6.4 ± 3.0. After the first month, the migraine days were reduced to 1.5 ± 1.5 with a mean difference of 5.0 ± 2.8 (*p*-value < 0.0001). The chronic migraine patients showed a mean of 16.1 ± 3.6 monthly migraine days before treatment. After the first month, the migraine days were reduced to 6.0 ± 4.6 with a mean difference of 10.0 ± 4.6 (*p*-value < 0.0001). The medication overuse headache patients had a mean of 13.2 ± 5.5 monthly migraine days before treatment. After the first month, the migraine days were reduced to 6.5 ± 6.5 with a mean difference of 6.3 ± 5.2 (p-value < 0.001), Fig. 2.

In this study, reported clinically relevant improvement of headaches were defined as at least 50% reduction of migraine days per month after at least 3 monthly doses of erenumab in comparison to the average of the 2 months prior to first dose of erenumab. A total of 73 patients (80.2%) reported a clinically relevant reduction in headaches. To identify patients who responded to treatment, we analyzed the association between different patient characteristics and their treatment result (Table 2). The suggested associations were confirmed by logistic regression (Table 3). Patients with episodic migraine (87.9%) showed the most improvements, followed by chronic migraine (84.1%) and medication overuse headache (50.0%). In logistic regression with episodic migraine as reference category, medication overuse headache showed a lower probability of therapy success with erenumab (odds ratio: 0.126, *p* = 0.039). Among the other patient characteristics, ethnicity (*p*-value = 0.015) and older age (p-value = 0.035) were associated with clinically relevant improvement of symptoms. Middle Eastern ethnicity was inversely related with improvement of symptoms, while older ages were associated with better treatment responses. In logistic regression with Middle Eastern ethnicity as reference category, Europeans were more likely to benefit from erenumab therapy (odds ratio: 12.788, *p* = 0.037). Patients from 31 to 40 years (77.8% response rate) and 41–65 years (89.9% response rate) benefited most from the treatment with the new CGRP receptor inhibitor. This suggested association of older ages and better treatment response was confirmed by logistic regression (*p* = 0.047). Neither gender nor dose increase of erenumab showed an association with the reported clinically relevant improvement of the symptoms.

**Table 2:** Response rate across the different patient characteristics

	Clinically relevant reduction of migraine days in all migraine types (≥50%)		
Variable	Yes (*n* = 73)	No (*n* = 18)	P-value
Gender			0.747
Female	63 (80.8%)	15 (19.2%)	
Male	10 (76.9%)	3 (23.1%)	
Range of age			0.035
0–20	1 (50.0%)	1 (50.0%)	
21–30	8 (57.1%)	6 (42.9%)	
31–40	21 (77.8%)	6 (22.2%)	
41–65	43 (89.9%)	5 (10.4%)	
Ethnicity			0.015
Middle East	30 (66.7%)	15 (33.3%)	
Asian	16 (94.1%)	1 (5.9%)	
Europe	19 (95.0%)	1 (5.0%)	
Rest of the world	8 (88.9%)	1 (11.1%)	
Type of migraine			0.008
Medication overuse headache	7 (50.0%)	7 (50.0%)	
Episodic migraine	29 (87.9%)	4 (12.1%)	
Chronic migraine	37 (84.1%)	7 (15.9%)	
Dose increase			0.369
Yes	10 (71.4%)	4 (28.6%)	
No

**Table 3:** Logistic regression for age, ethnicity, and type of migraine

Variable	Regression coefficient	Standard error	P-value	Odds ratio	95% confidence interval
Age	0.057	0.029	0.047	1.059	1.001–1.120
Ethnicity					
Middle East			0.035		
Asian	2.220	1.205	0.065	9.208	0.868–97.693
Europe	2.549	1.221	0.037	12.788	1.168–140.001
Rest of the world	2.283	1.401	0.103	9.805	0.630–152.644
Type of migraine					
Episodic migraine			0.083		
Chronic migraine	−0.296	0.786	0.706	0.744	0.159–3.470
Medication overuse headache	−2.071	1.004	0.039	0.126	0.018–0.902

Throughout this study, 84.6% were treated monthly. In a subset (15.4%), the dose was increased to 140 mg per month. 9 patients received 140 mg erenumab monthly (2 × 70 mg injections), whereas 5 patients had one injection every two weeks. In these patients there was a trend towards improvement of headaches. In terms of side effects, only minor non-treatment limiting side effects were reported. These included 1 patient with hair loss, 1 patient with fatigue symptoms, 3 patients with skin reactions, and 2 patients with initial headache after injection. Among the 58 patients who were excluded from the analysis because of the study requirements of at least 3 consecutive monthly injections of erenumab over ≥3 months of follow-up, 7 patients discontinued the medication due to side effects. The remaining patients were lost to follow-up, specific reasons for which were unknown.

## Discussion

To our knowledge, no post-marketing studies regarding the use of CGRP receptor antibody erenumab have been published from the Middle East region. The results of this study suggest a robust effect of this new preventive treatment in patients with three types of migraine headaches. In this post-marketing cohort, clinically relevant improvement of headaches (≥50% reduction) in overall migraine types was observed in 80.2% of the patients. This outcome is higher than the percent reduction achieved in previous randomized controlled trials, which showed that 36–43.3% of patients receiving erenumab for the three migraine types achieved 50% or greater reduction in the mean number of migraine days per month [13–15].

The clinically relevant improvement of headaches was associated with the type of migraine. We found the most favorable responses occurred in patients with episodic migraine, followed by chronic migraine and medication overuse headache. In contrast to the randomized controlled trials, revealing a slope of improvement over 6 months, a benefit was achieved and maintained after the first dose.

In our study, episodic migraine seemed to have the most benefit where 87.9% of the patients reported clinically relevant improvement of headaches. Our results show a mean difference of 5.0 ± 2.8 monthly migraine days (78% reduction) in comparison to baseline. In contrast, Goadsby et al. showed that episodic migraine treated with 70 mg erenumab had a success rate of 43.3%. The number of monthly migraine days was reduced by 3.2 ± 0.2. The 140 mg erenumab dose led to a 50.0% success rate and a reduction of 3.7 ± 0.2 days per month [15].

With regard to chronic migraine, 84.1% of patients reported a clinically relevant improvement of headaches. Likewise, in the randomized controlled trial by Tepper et al., erenumab reduced the frequency of migraine and the number of medications in patients with chronic migraine [13]. In 2017, Tepper et al. reported that 40% (70 mg erenumab) and 41% (140 mg erenumab) of the patients with chronic migraine perceived a clinically relevant improvement and their migraine days were reduced in both by 6.6 ± 0.4 days per month in both groups [14]. A recent study studied the effectiveness of erenumab on migraine-induced events, including outpatient visits, hospitalizations, or ER visits. After 6 months, the mean number of events decreased from 1.03 to 0.77 (rate ratio: 0.75, 95% CI: 0.71–0.79, *P*-value < 0.0001) [16].

For medication overuse headache, our results suggest a reduction of migraine days with a mean difference of 6.3 ± 5.2 (47.8% reduction), and 50.0% of patients reported a clinically relevant improvement of their headaches. Anti-CGRP monoclonal antibodies could be prescribed either simultaneously with medication withdrawal or even before starting withdrawal [17]. A recent randomized controlled trial showed clinically relevant improvement in patients with medication overuse headaches receiving erenumab. Groups receiving 70 and 140 mg of erenumab showed respective ≥50% responder rates of 36 and 35%, respectively. The reduction of monthly migraine days amounted to 6.7 ± 1.1 days in the 140 mg dose group [13]. Another study observed the effectiveness of anti-CGRP monoclonal antibodies, including erenumab, on patients with medication overuse headaches. After six months of treatment, 63.6% of patients had a response rate ≥ 50% in migraine days per month and 53.5% showed the same improvement in headache days per month [18].

In addition, we analyzed various patient characteristics to predict treatment responses. Treatment success was less likely in younger than older patients in this study. This age-related response has not been described in other studies before. While it may simply reflect a finding resulting from the small sample size, an increase of therapeutic response was shown in randomized trials of acute migraine treatments where the rates of pain relief and pain-free status were also inversely related to age. These findings were interpreted to indicate an increasing therapeutic gain over placebo with age [19]. Given the relatively high treatment responses to placebo in all randomized controlled trials on preventive treatment with erenumab, similar relationships may exist. To further test this hypothesis, the analysis of age and treatment response should be further investigated. In the randomized controlled trial [15] a powerful effect of placebo on episodic migraine was established. However, the relationship between age and treatment response has not been reported in this trial.

We also observed variable responses to erenumab over various ethnicities in this cohort study. These findings may be cultural, coping, and/or adherence to medication [20]. Alternatively, genetic disposition with differing pain perception could also be at play [21]. No gender influence on treatment response could be found in this study and only a few patients were offered dose increases. Although these patients tended to report decreased headaches as suggested in previous randomized trials further studies need to address this issue [13–15].

In this study, erenumab was well tolerated. Only few patients had side effects during this study follow-up period. These findings are also in line with the results of randomized controlled trials [15]. While there is no safety concern in any reported study so far including our investigation, the expected high penetrance of CGRP receptor inhibitors in the market should be monitored and careful pharmacovigilance is recommended [22].

It is noteworthy that 58 patients (38.8%) out of our original patient population (149 patients) did not meet the study requirements of at least 3 consecutive monthly injections of erenumab over ≥3 months of follow-up and were therefore excluded from the analysis. This relatively high number requires further clarification. Seven patients discontinued the medication due to side effects. The remaining patients were lost to follow-up, specific reasons for which were unknown. We speculate that problems with coverage by patient’s health insurance, changing healthcare providers (e.g. hospital or city), moving away, or complete treatment response are possible reasons. Alternatively, patients may have experienced remission of symptoms and decided to suspend further visits.

As a limitation with all observational, in particular retrospective studies, treatment effects may have been overestimated and possible confounders, such as recall bias, inconsistent documentation, or missing data impaired the results. This study is also limited by a relatively small sample size since it was conducted in a single center with limited number of patients. Multicentric studies, with large sample size populations, are required to further validate our results in the future.

In conclusion, this retrospective observational study in the early era of monoclonal CGRP receptor inhibitors shows a significant effect of erenumab in the treatment of all migraine types. Erenumab led to a clinicallyrelevant improvement of headaches and a reduction of monthly migraine days within the first few months of treatment. The results suggest a relationship between age, ethnicity, and treatment response that needs to be evaluated in further studies. Further research is required to address adherence and effectiveness among various age groups and ethnicities.

## Data Availability

The datasets used and/or analyzed during the current study are available from the corresponding author on reasonable request.

## Abbreviations

CGRP: Calcitonin-gene related peptide
ICHD-3: International Classification of Headache Disorders
NSAIDs: Non-steroidal anti-inflammatory drugs
UAE: United Arab Emirates
SPSS: Statistical Package for the Social Sciences
